# Time kinetics of physical activity, sitting, and quality of life measures within a regional workplace: a cross–sectional analysis

**DOI:** 10.1186/s12889-016-3487-x

**Published:** 2016-08-15

**Authors:** Daniel B. Lindsay, Sue Devine, Rebecca M. Sealey, Anthony S. Leicht

**Affiliations:** 1 Psychology and Public Health, James Cook University, Townsville, QLD Australia; 2 Public Health, James Cook University, Townsville, QLD Australia; 3 Sport and Exercise Science, James Cook University, Townsville, QLD 4811 Australia

**Keywords:** Sedentary behaviour, Occupation, University, Walking, Gender, Employment

## Abstract

**Background:**

Interventions to increase physical activity and reduce sedentary behaviours within the workplace have been previously investigated. However, the evolution of these constructs without intervention has not been well documented. This retrospective study explored the natural progression or time kinetics of physical activity, sedentary behaviours and quality of life in a professional skilled workplace where focussed interventions were lacking.

**Methods:**

Participants (*n* = 346) employed as full-time staff members at a regional university completed an online survey in 2013 assessing physical activity and sedentary behaviours via the International Physical Activity Questionnaire, and quality of life via the Short-Form 36v2 questionnaire. Differences between that cohort of participants and an initial sample of similar participants (2009, *n* = 297), accounting for gender and staff categories (academic vs. professional), were examined using ANCOVAs with working hours as a co-variate.

**Results:**

In comparison to the initial cohort, the follow-up cohort reported significantly less leisure-time, total walking, total vigorous and total physical activity levels, and lower overall physical health for quality of life (*p* < 0.05). In contrast, the follow-up cohort reported a significantly greater weekly sitting time, greater mental health scores for quality of life and greater total moderate physical activity levels (*p* < 0.05) compared to the initial cohort.

**Conclusions:**

Over a 4-year timeframe and without focussed workplace interventions, total physical activity levels were lower with sedentary behaviours greater at a rate twice that reported previously. Continuation of these undesirable health behaviours may impact negatively on worker productivity and health at a greater rate than that currently reported. Workplace interventions targeting sedentary behaviours and physical activity should be actively incorporated into organisations to counteract the alarming behavioural trends found in this study to maintain and/or enhance employee health and productivity.

## Background

Lower levels of physical activity (PA) and increased sedentary time have contributed to an increase in negative health outcomes including obesity and diabetes [1]. Both behaviours have been identified as important and independent risk factors for premature mortality [2–4]. For example, a 2 % increase in all-cause mortality risk was associated with each hour of daily sitting, even when PA was accounted for in the analysis [2]. This risk was enhanced when daily sitting exceeded 7 h per day [2], a value similar to that seen within the workplace [5–7]. Likewise, low levels of PA, particularly moderate-vigorous PA, was associated with greater all-cause mortality (risk ratio of 3.3) that was enhanced when combined with greater sitting time [4]. Low PA and high levels of sedentary behaviour may be particularly relevant to the workforce with workplace sitting accounting for a majority of sitting time during a weekday [5, 6]. Additionally, high amounts of workplace sitting appear to result in high levels of leisure-time sitting [5, 8]. Therefore, the workplace provides a key environment for PA and sedentary behaviour management to impact on health and well-being.

Working adults comprise a significant proportion of the population [9]. Therefore, it is important for health promotion efforts to focus on this particular group for long-term health benefits [10]. Previously, PA and/or sedentary behaviours were examined within a university setting due to the substantial number of working hours completed by employees and limited time to undertake PA, a significant barrier reported for PA participation [7, 11–14]. This setting exemplifies the typical white collar workplace where there has been a focus on employee productivity and/or health [15–19] via examination of PA, sedentary behaviours, and quality of life (QOL) [11, 12, 20–22].

To our knowledge, most studies of health, PA and sedentary behaviour within the workplace, including universities, have focussed on specific interventions in small sub-populations of employees over a short timeframe [11, 12, 23, 24]. Very few have examined PA and sedentary behaviours within a workforce over time *without* targeted interventions. A greater understanding of the typical development of these factors will provide a comparative reference for future workplace interventions to gauge success. Distinctively, the natural progression of sedentary behaviours was examined in the Danish workforce between 1990 and 2010 with sitting time increased by 18 % in individuals of a high socio-economic status [25]. This long-term examination highlighted an increase of sedentary behaviour for a select group that equated to approximately 0.9 % annually, based upon a linear progression [25]. Recent emphasis on workplace interventions suggests a much greater development rate for sedentary behaviour that remains to be confirmed, possibly via a shorter term examination [13, 18, 26]. Further, the results of van der Ploeg and colleagues focused on the sedentary behaviours of a subsample of the working population, with PA and accompanying QOL not examined [25]. Investigation of the normal progression of PA and sedentary behaviour over a reasonable timeframe may clarify the true kinetics of these factors and their impact on health (e.g. QOL) within the workplace. Therefore, the aim of the current study was to examine the change in PA, sedentary behaviour and QOL over time *without* focussed interventions in a professional skilled workplace (i.e. university). It was hypothesised that PA levels and QOL would decrease over time while sedentary behaviours would increase as a natural progression within the workplace [25].

## Methods

### Participants

This study used convenience sampling in an attempt to recruit all staff employed full-time at a regional university in Australia as previously reported [7]. Due to the transient nature of employees within regional and rural/remote Australia [27], a cross sectional observation design was employed with surveys conducted at two time-points, 2009 and 2013. Results from the initial survey and cohort were previously reported [7], with the current study exploring the transient changes (i.e. four years later). For the follow-up cohort, full-time staff employed within the organisation were invited to participate via email and were directed to a secure web site to complete a self-administered survey of approximately 25 min duration.

Of the eligible, full-time, university staff (*N* = 2028), 346 (17.1 %) volunteered to participate in this online study with 113 (~33 %) participants identified as academics and 233 (~67 %) identified as professionals. The academic and professional distinction was made based on participant’s primary role at the university with academics primarily involved with teaching and/or research, and professionals primarily involved with administrative, governance or technical issues [7].

### Procedures

The online survey assessed demographic characteristics via specific questions about staff’s age, height, mass, gender, employment type and number of hours worked per typical working week. Additionally, the survey assessed participants’ QOL via the Short-Form 36 (SF-36) version 2 [28], PA levels during the past seven days using the International Physical Activity Questionnaire (IPAQ) long version (www.ipaq.ki.se), and other factors (e.g. barriers/motivators to exercise) that were not considered in the current study (data not shown).

The SF-36 is a valid and reliable tool for the assessment of QOL [29] and consists of 8 health domains including physical functioning (PF), role-physical (RP), bodily pain (BP), general health (GH), vitality (VT), social functioning (SF), role-emotional (RE) and mental health (MH) [28]. Responses to questionnaire items were summed and scores transformed using Australian population norms [30], with normative-based scores calculated between 0 (worst health) and 100 (best health). Additionally, summary measures of overall physical (PCS) and mental (MCS) health were calculated using the health domain scores [28].

The IPAQ is a valid and reliable tool for measuring PA levels of adults between the age of 18 and 69 years [31] and includes questions about physical activities in the domains of work, transport, yard/garden and leisure with results presented in MET-minutes per week [32]. Additionally, the IPAQ identified daily and total week sitting time [7] that has been moderately correlated with objective measures of sitting [33, 34]. The IPAQ has been utilised extensively as an outcome tool in studies of PA, sedentary behaviour and QOL [35–38], including those examining workplaces [7].

### Statistical analysis

All data were analysed using the Statistical Package for the Social Sciences (IBM SPSS Statistics for Windows, Version 22.0. Armonk, NY: IBM Corp) with data normality assessed using the Kolmogorov-Smirnov statistic. Data were presented as mean ± standard deviation where appropriate. Significant differences between staff classifications (academic vs. professional), genders (male vs. female) and cohorts (initial vs. follow-up) were determined using 2 × 2 ANCOVAs with hours worked as a co-variant. Staff classification and gender were examined within the current study as both factors were reported to influence PA and QOL levels within a university workplace [7]. A mean difference (MD) score was calculated to easily demonstrate the cohort differences (i.e. change in variable over time). This MD score was calculated by subtracting the initial cohort scores from the follow-up cohort scores with positive scores indicating an increase over time and negative scores indicating a decrease in the relevant variable. Pearson correlation coefficients were calculated to assess the association between demographic variables, PA, QOL and sedentary behaviour. A *p*-value of <0.05 was set as the level of significance for all analyses.

## Results

The age (43.2 ± 11 years), height (169.6 ± 9.1 cm), mass (73.3 ± 15.8 kg), gender distribution (29.4 % male, 70.6 % female), employment type (32.7 % academic, 67.3 % professional) and hours worked (43.8 ± 10.0) for the follow-up cohort were not significantly different to the initial cohort (*p* > 0.05), indicating similar demographics for both cohorts [7].

The PA levels of academic and professional staff are presented in Table 1. Compared to the initial cohort, the follow up cohort undertook significantly less walking and total transport PA, significantly less vigorous and total leisure PA and significantly less total walking, vigorous and total PA (Table 1). All staff in the follow-up cohort significantly exhibited greater total moderate PA (Table 1). In the follow-up cohort, professional staff undertook significantly less moderate PA at work, significantly more yard work/chores across all categories, and significantly more total moderate PA compared to academic staff (Table 1).

**Table 1:** Mean ± SD and change from initial cohort for physical activity and sitting for staff of the follow-up cohort

	Follow-up cohort			MD		
	Academic (*n* = 96)	Professional (*n* = 213)	All (*n* = 309)	Academic (*n* = 106)	Professional (*n* = 158)	All (*n* = 264)
Work (MET-minutes per week)						
Walking	60 ± 177	63 ± 177	62 ± 176	−160^†^	−76	−112
Moderate activity	223 ± 1143	139 ± 592^*^	167 ± 817	136^†^	−3	49
Vigorous activity	214 ± 875	184 ± 900	194 ± 891	111	−2	43
Total	496 ± 1885	386 ± 1438	423 ± 1599	87	−81	−20
Transport (MET-minutes per week)						
Walking	86 ± 136	128 ± 275	114 ± 238	−205^†^	−249^†^	−227^†^
Cycling	236 ± 633	193 ± 713	207 ± 687	3	−124	−74
Total	322 ± 656	321 ± 765	321 ± 732	−202^†^	−373^††^	−301^††^
Yard/garden (MET-minutes per week)						
Vigorous yard chores	326 ± 643	556 ± 951^*^	480 ± 867	15	−9	23
Moderate yard chores	460 ± 667	672 ± 796^*^	602 ± 761	−1	207^†^	139
Moderate inside chores	326 ± 563	512 ± 618^*^	450 ± 606	−6	95	69
Total	1095 ± 1327	1698 ± 1656^*^	1500 ± 1581	−9	251^†^	199^†^
Leisure (MET-minutes per week)						
Walking	394 ± 467	323 ± 445	347 ± 453	39	29	27
Moderate activity	289 ± 369	329 ± 486	315 ± 450	100	84	94
Vigorous activity	144 ± 296	186 ± 363	172 ± 342	−594^††^	−472^††^	−520^††^
Total	827 ± 764	838 ± 940	834 ± 884	−455^††^	−359^††^	−399^††^
Total physical activity (MET-minutes per week)						
Walking	540 ± 510	514 ± 589	522 ± 563	−326^††^	−297^††^	−312^††^
Moderate activity	1859 ± 2182	2400 ± 2008^*^	2220 ± 2080	246^†^	250^†^	298^†^
Vigorous activity	358 ± 913	370 ± 981	366 ± 958	−483^††^	−475^††^	−477^††^
All	2757 ± 2922	3284 ± 2780	3109 ± 2848	−563^††^	−522^††^	−491^††^
Sitting (minutes)						
Total per week	3214 ± 1041	2988 ± 965	3064 ± 995	210^††^	229^††^	201^††^
Total per day	459 ± 148	427 ± 138	438 ± 142	30	33	29

*MD* absolute mean difference from initial cohort

^*^ *p* < 0.05 vs. academic; ^†^ *p* < 0.05 & ^††^ *p* < 0.01 vs. initial cohort

The PA levels of male and female staff are presented in Table 2. Compared to the initial cohort, both male and female staff undertook significantly less walking as transport, significantly less vigorous and total leisure PA, total walking, total vigorous and total PA with significantly more total moderate PA (Table 2). In the follow-up cohort, male staff undertook significantly more walking and total PA at work, significantly more cycling and total transport, significantly more moderate leisure PA with significantly less moderate yard and inside chores compared to females (Table 2).

**Table 2:** Mean ± SD and change from initial cohort for physical activity and sitting for staff of the follow-up cohort

	Follow-up cohort		MD	
	Male (*n* = 98)	Female (*n* = 233)	Male (*n* = 73)	Female (*n* = 130)
Work (MET-minutes per week)				
Walking	91 ± 216	50 ± 157^*^	−59	−135
Moderate activity	197 ± 780	156 ± 837	70	43
Vigorous activity	216 ± 1014	186 ± 839	−88	113
Total	503 ± 1530	393 ± 1635^**^	−79	21
Transport (MET-minutes per week)				
Walking	141 ± 282	103 ± 218	−216^†^	−229^†^
Cycling	321 ± 739	161 ± 663^*^	40	−120
Total	463 ± 776	263 ± 709^*^	−176	−351^††^
Yard/garden (MET-minutes per week)				
Vigorous yard chores	518 ± 753	468 ± 914	−32	58
Moderate yard chores	481 ± 639	657 ± 803^*^	107	148
Moderate inside chores	223 ± 285	548 ± 677^**^	−18	96
Total	1185 ± 1316	1644 ± 1664^**^	20	274^†^
Leisure (MET-minutes per week)				
Walking	297 ± 400	368 ± 475	12	30
Moderate activity	388 ± 522	283 ± 415^*^	212^†^	39
Vigorous activity	206 ± 383	160 ± 325	−651^††^	−448^††^
Total	890 ± 904	811 ± 880	−428^††^	−379^††^
Total physical activity (MET-minutes per week)				
Walking	529 ± 551	521 ± 571	−263^†^	−335^††^
Moderate activity	2127 ± 1680	2273 ± 2232	377^††^	264^†^
Vigorous activity	421 ± 1076	346 ± 909	−741^††^	−336^††^
Physical activity	3077 ± 2387	3140 ± 3030	−628^††^	−406^††^
Sitting (minutes)				
Total per week	3101 ± 1123	3052 ± 938	232^††^	192^†^
Total per day	443 ± 161	436 ± 134	33	27

MD absolute mean difference from initial cohort

^*^ *p* < 0.05 & ^**^ *p* < 0.01 vs. male; ^†^ *p* < 0.05 & ^††^ *p* < 0.01 vs. initial cohort

In regards to sitting, the follow-up cohort experienced greater weekly sitting time compared to the initial cohort with similar differences noted for both employment types and genders (Tables 1 and 2).

There were no differences in QOL noted between employment types or between genders within the follow-up cohort (Tables 3 and 4). Compared to the initial cohort, academic staff exhibited greater VT, RE, MH and MCS, and reduced BP and PCS (Table 3). Similarly, professional staff exhibited greater VT and MCS with reduced BP and PCS compared to the initial cohort (Table 3). With respect to gender, male staff exhibited greater MCS and reduced PF, BP and PCS compared to the initial cohort (Table 4). Female staff also exhibited greater VT and MCS with reduced BP and PCS compared to the initial cohort (Table 4).

**Table 3:** Mean ± SD and change from initial cohort for quality of life for staff of the follow-up cohort

	Follow-up cohort			MD		
	Academic (*n* = 96)	Professional (*n* = 213)	All (*n* = 309)	Academic (*n* = 106)	Professional (*n* = 158)	All (*n* = 264)
PF	53.1 ± 6.0	52.4 ± 7.2	52.6 ± 6.8	−0.4	−0.5	−0.5
RP	52.9 ± 5.2	52.8 ± 5.5	52.8 ± 5.4	0.1	−0.2	−0.1
BP	48.5 ± 7.9	47.7 ± 7.0	47.9 ± 7.3	−2.5^*^	−2.3^*^	−2.5^*^
GH	50.2 ± 10.0	48.7 ± 7.4	48.7 ± 9.5	0.0	−0.8	−1.1
VT	48.1 ± 7.1	47.2 ± 7.7	47.5 ± 7.5	4.5^**^	1.9	2.9^*^
SF	49.4 ± 9.9	49.3 ± 9.7	49.3 ± 9.7	0.3	0.1	0.1
RE	51.0 ± 5.8	50.0 ± 6.8	50.1 ± 6.5	3.7^**^	0.6	1.5
MH	48.6 ± 9.6	48.8 ± 9.8	48.8 ± 9.7	2.6^*^	0.4	1.4
PCS	51.2 ± 5.5	50.4 ± 5.8	50.6 ± 5.7	−4.8^**^	−3.8^**^	−4.3^**^
MCS	49.3 ± 6.7	48.7 ± 7.0	48.9 ± 6.9	5.7^**^	2.2^*^	3.5^**^

*MD* absolute mean difference from initial cohort, *PF* physical functioning, *RP* role-physical, *BP* bodily pain, *GH* general health, *VT* vitality, *SF* social functioning, *RE* role-emotional, *MH* mental health, *PCS* overall physical health summary, *MCS* overall mental health summary

^*^ *p* < 0.05 & ^**^ *p* < 0.01 vs. initial cohort

**Table 4:** Mean ± SD and change from initial cohort for quality of life for staff of the follow-up cohort

	Follow-up cohort		MD	
	Male (*n* = 88)	Female (*n* = 219)	Male (*n* = 95)	Female (*n* = 169)
PF	51.2 ± 8.6	53.1 ± 5.9	−2.2^*^	0.2
RP	52.3 ± 6.1	53.0 ± 5.1	−1.0	0.3
BP	47.2 ± 8.2	48.2 ± 7.0	−3.4^**^	−2.1^*^
GH	48.7 ± 9.5	49.4 ± 9.7	−0.7	−0.5
VT	47.0 ± 8.0	47.8 ± 7.3	1.2	3.8^**^
SF	49.4 ± 10.4	49.2 ± 9.6	−0.3	0.3
RE	49.5 ± 6.7	50.3 ± 6.5	0.4	2.0
MH	48.3 ± 9.6	49.0 ± 9.8	0.1	2.0
PCS	49.8 ± 6.7	50.9 ± 5.3	−5.2^**^	−4.0^**^
MCS	48.5 ± 7.2	49.1 ± 6.8	2.3^*^	4.2^**^

*MD* absolute mean difference from initial cohort, *PF* physical functioning, *RP* role-physical, *BP* bodily pain, *GH* general health, *VT* vitality, *SF* social functioning, *RE* role-emotional, *MH* mental health, *PCS* overall physical health summary, *MCS* overall mental health summary

^*^ *p* < 0.05 & ^**^ *p* < 0.01 vs. initial cohort

The number of hours worked for all staff in the follow-up cohort was significantly associated with age (*r* = 0.188, *p* < 0.01), RE (*r* = 0.125, *p* < 0.05) and total sitting time (*r* = 0.249, *p* < 0.01). Total sitting time was significantly and negatively associated with RP (*r* = −0.115, *p* < 0.05), BP (*r* = −0.124, *p* < 0.05), MH (*r* = −0.173, *p* < 0.01), PCS (*r* = −0.133, *p* < 0.05) and MCS (*r* = −0.116, *p* < 0.05). Relationships were maintained with similar correlation coefficients when analysed separately for employment category and gender. No other significant correlations were noted.

## Discussion

Using a cross-sectional design, the current study described changes in PA levels, sitting behaviour and QOL in a regional workplace by comparing two different cohorts. Four years later, lower PA levels (leisure, walking, vigorous and total) and overall physical health (QOL) were observed indicating a potential decrease in PA and QOL over time. The lack of focussed interventions within a white-collar workplace may lead to undesirable changes in several risk factors for employee well-being. Development of the most appropriate and effective workplace interventions to address these alarming transformations are vital to minimise further deterioration and subsequent declines in employee health and productivity.

The current results were based upon cross-sectional analyses and self-report methods of PA from two different cohorts that may be limited in terms of reliability, validity and recall bias [39, 40]. Therefore, the current results should be interpreted with some caution. Nonetheless, the most crucial finding of the current study was the significant change in total weekly sitting time with an increase of ~200 min per week (half an hour per day) over the 4-year period. This increase was ~2 % per year (assuming a linear trend) and twice that reported for the Danish workforce between 1990 and 2010 [25]. This trend was disturbing given the relatively short timeframe of four years and the growing body of evidence about the negative health impacts of prolonged sitting [2, 4, 6, 8]. Comparatively, this 30-min increase of sitting per day could equate to ≥1 % increase in all-cause mortality risk [2]. Our results highlight an escalated rate of sedentary behaviour within the workplace and an emerging serious risk factor for workers and management that requires attention.

Importantly, the sitting time increase in the current study was independent of employment category (and indirectly tasks/duties) and gender confirming the workplace as an increasingly sedentary-based environment for all workers [41, 42]. Given the strong, positive relationships between workplace and non-workplace sedentary behaviours [5, 43], and sedentary behaviours and cardiovascular/metabolic disease risk [6, 44, 45], the current results provide further evidence of the workplace being a critical setting for sedentary behaviour reduction and improved employee health and well-being. Further, incorporation of PA may provide additional benefits including increased employee productivity [19, 46–48]. Therefore, interventions targeting both sedentary behaviours and changes in lifestyle, such as increased PA, may be essential to support employee productivity, minimise health risks and to counteract the evolving sedentary workplace [49].

Another concerning trend found in the current sample was the reduction in PA levels within most categories (i.e. leisure, walking, vigorous and total PA) over time. A similar reduction in PA was recently reported in a cross-sectional survey of Czechoslovakian adults during 2002 to 2011 [50]. Paired with the increased sitting time, the reduction in PA levels places the current workers at an increased risk of premature mortality [5, 41, 42]. This PA reduction could have simply reciprocated the increase in sitting time. However, total moderate PA was greater (10.4–17.7 %) for the follow-up cohort which may have reflected a compensatory effort by participants to enhance PA given their level of workplace sitting [51], or a renewed effort to undertake moderate PA daily in accordance with updated PA recommendations [52]. Regardless, this increase in moderate PA was at the expense of reductions in other PA categories, in particular total self-reported PA, and an increased sitting time. Several studies have recommended sitting time as the key focus for health interventions with PA of lesser importance [43, 53]. Others though have highlighted the importance of PA contributions in addition to sitting time for improved health [2, 54]. The current, long-term data would indicate that both sitting and PA foci require attention to support the well-being of employees [49].

Interventions aimed at interrupting sitting behaviour have been shown to reduce sitting time [49, 55–57], enhance PA levels [51, 58], and improve cardio-metabolic risk factors [48, 59]. In a recent systematic review, sedentary behaviour interventions were concluded to be effective in reducing workplace sitting for white-collar workers with multi-component and environmental based approaches particularly effective [49]. Others utilising workplace PA interventions have reported similar benefits including improvements in QOL or well-being [20, 60, 61]. In the current study, the absence of a workplace intervention may have contributed to a lower overall physical health measure of QOL for the follow-up cohort. This lower QOL may have resulted from the increased sitting levels, lower total PA levels and/or potentially poorer fitness of employees. Fitness levels of employees were not assessed in the current study but may be a direct result of changes in sitting and PA behaviours [12, 62]. Examination of fitness along with sitting and PA behaviours may clarify the importance of these factors individually or in combination for employee health and productivity [12, 63].

Despite a poorer physical QOL, the follow-up cohort exhibited greater overall mental health (i.e. MCS). This was unexpected given the similarity in demographics between cohorts, the lower PA and greater sedentary behaviour of the follow-up cohort, and the significant and negative associations between mental health indices (MH and MCS) and total sitting time for the initial and follow-up cohorts [7]. An explanation for this result was not obvious but may be related to the greater moderate PA levels for the follow-up cohort with moderate intensity PA demonstrated to enhance mental well-being [20, 22]. Future studies may elucidate the relative contributions of sedentary behaviour and PA on both physical and mental health of employees including changes with workplace PA and/or sitting interventions. These studies are vital given the substantial number of working hours undertaken by employees.

Previously, we reported significant relationships between working hours, sitting time and QOL in university employees [7]. Similar relationships were again reported in the current study, as well as in a separate study where substantial working hours were associated with decreased QOL for accomplished health professionals [64]. This relationship likely reflects the impact of working hours on sitting time, which later influences a range of metabolic and cardiovascular risk factors and finally QOL [6, 43]. Several studies have reported on the beneficial effects of reducing sitting time for cardiovascular biomarkers and risk [48, 59]. However, the optimal workplace intervention to reduce sitting behaviour, increase PA levels and enhance employee health remains unknown [65]. As indicated beforehand, several workplace interventions have been undertaken with reported benefits for PA and/or sitting levels [20, 55, 56, 60, 61]. Future studies will elucidate the benefits of workplace interventions to counteract the developing, sedentary work environment, an important contributor to employee health [66].

It is worth noting that the findings from the current study were limited to cross-sectional sampling over time and a small proportion of staff from one workplace (<20 %). As stated previously, the examination of different cohorts may have inherent limitations concerning survey responses. Though, there were no significant differences in demographics for the two cohorts, indicating comparable population samples. Despite this similarity, a degree of caution is recommended in interpreting the changes in PA and sitting behaviour for the current study. Possible confounders such as working hours were considered in the current study however, others such as fitness levels, cognitive function, work environment, etc. were not and may have influenced results. Further, participants were from the same workplace and surveyed at the same time of the year, across cohorts, to minimise the influences of annual workplace activity and/or season. Longitudinal sampling, using objective measures of PA and sitting across a variety of workplaces may confirm the precise time kinetics of PA and sitting behaviour. Secondly, this study examined staff from one organisation with the proportion of the entire staff who participated in this survey, smaller than expected. Therefore, the current results may not be reflective of all staff within multiple organisations. However, the current study examined a larger sample than prior studies [15, 67] with results for sedentary behaviour similar to that of multiple worker groups within the Danish workforce [25]. Nonetheless, future studies are encouraged to enrol larger working populations and from a range of workplaces for increased generalisability of results to the working population. Finally, all measures in this study were self-reported with the IPAQ previously suggested to overestimate PA in some populations [68]. The limitations of self-reported PA levels have been well documented [39, 40] with the use of objective assessments (e.g. accelerometry) recommended to confirm the current results and clarify the kinetics of PA and sitting behaviour within the workplace.

## Conclusions

Using a cross-sectional design, the current study has highlighted the natural progression of PA, sitting behaviour and QOL in a regional workplace over a 4-year timeframe *without* focussed intervention. Importantly, the lack of a focussed workplace intervention may have contributed to a rapid increase in sedentary behaviour, and decreases in most categories of PA and physical QOL within a professional skilled workplace. Despite the potential health risks associated with such behaviours, the current developing nature of the workplace may limit individuals’ ability to reduce their sitting or increase their PA levels without the assistance of focussed interventions. Greater focus on workplace interventions including optimal activities, frequency, duration and intensity may assist in the reduction of risk factors and improvement of employee health and/or productivity.

## Abbreviations

PA: Physical activity
QOL: Quality of life

## References

[1] Australian Institute of Health and Welfare. Australia’s Health 2010. Canberra: Australian Institute of Health and Welfare; 2010.

[2] Chau JY, Grunseit AC, Chey T, Stamatakis E, Brown WJ, Matthews CE (2013). Daily sitting time and all-cause mortality: a meta-analysis. PLoS One.

[3] Samitz G, Egger M, Zwahlen M (2011). Domains of physical activity and all-cause mortality: systematic review and dose–response meta-analysis of cohort studies. Int J Epidemiol.

[4] Schmid D, Ricci C, Leitzmann MF (2015). Associations of objectively assessed physical activity and sedentary time with all-cause mortality in US adults: the NHANES study. PLoS One.

[5] Saidj M, Menai M, Charreire H, Weber C, Enaux C, Aadahl M (2015). Descriptive study of sedentary behaviours in 35,444 French working adults: cross-sectional findings from the ACTI-Cités study. BMC Public Health.

[6] Mummery WK, Schofield GM, Steele R, Eakin EG, Brown WJ (2005). Occupational sitting time and overweight and obesity in Australian workers. Am J Prev Med.

[7] Leicht AS, Sealey RM, Devine S (2013). Relationship between employment category and gender on quality of life, physical activity and their barriers and motivators, for full-time university staff. Int J Workplace Health Manag.

[8] Chau JY, van der Ploeg HP, Merom D, Chey T, Bauman AE (2012). Cross-sectional associations between occupational and leisure-time sitting, physical activity and obesity in working adults. Prev Med.

[9] Cahalin LP, Kaminsky L, Lavie CJ, Briggs P, Cahalin BL, Myers J (2015). Development and implementation of eorksite health and wellness programs: a focus on non-communicable disease. Prog Cardiovasc Dis.

[10] Chau JY, van der Ploeg HP, Dunn S, Kurko J, Bauman AE (2011). A tool for measuring workers’ sitting time by domain: the workforce sitting questionnaire. Br J Sports Med.

[11] Gilson ND, Puig-Ribera A, McKenna J, Brown WJ, Burton NW, Cooke CB (2009). Do walking strategies to increase physical activity reduce reported sitting in workplaces: a randomized control trial. Int J Behav Nutr Phys Act.

[12] Butler CE, Clark BR, Burlis TL, Castillo JC, Racette SB (2015). Physical activity for campus employees: a university worksite wellness program. J Phys Act Health.

[13] Cooper K, Barton GC (2015). An exploration of physical activity and wellbeing in university employees. Perspect Public Health.

[14] Booth ML, Bauman A, Owen N, Gore CJ (1997). Physical activity preferences, preferred sources of assistance, and perceived barriers to increased activity among physically inactive Australians. Prev Med.

[15] Chae D, Kim S, Park Y, Hwang Y (2015). The effects of an academic--workplace partnership intervention to promote physical activity in sedentary office workers. Workplace Health Saf.

[16] Harding J, Freak-Poli RL, Backholer K, Peeters A (2013). Change in health-related quality of life amongst participants in a 4-month pedometer-based workplace health program. J Phys Act Health.

[17] Merrill RM, Aldana SG, Garrett J, Ross C (2011). Effectiveness of a workplace wellness program for maintaining health and promoting healthy behaviors. J Occup Environ Med.

[18] Pereira MJ, Coombes BK, Comans TA, Johnston V (2015). The impact of onsite workplace health-enhancing physical activity interventions on worker productivity: a systematic review. Occup Environ Med.

[19] Puig-Ribera A, Martínez-Lemos I, Giné-Garriga M, González-Suárez ÁM, Bort-Roig J, Fortuño J (2015). Self-reported sitting time and physical activity: interactive associations with mental well-being and productivity in office employees. BMC Public Health.

[20] Freak-Poli RL, Wolfe R, Wong E, Peeters A (2014). Change in well-being amongst participants in a four-month pedometer-based workplace health program. BMC Public Health.

[21] Groeneveld IF, Proper KI, van der Beek AJ, Hildebrandt VH, van Mechelen W (2010). Lifestyle-focused interventions at the workplace to reduce the risk of cardiovascular disease--a systematic review. Scand J Work Environ Health.

[22] Milani RV, Lavie CJ (2009). Impact of worksite wellness intervention on cardiac risk factors and one-year health care costs. Am J Cardiol.

[23] Neuhaus M, Healy GN, Dunstan DW, Owen N, Eakin EG (2014). Workplace sitting and height-adjustable workstations: a randomized controlled trial. Am J Prev Med.

[24] Alkhajah TA, Reeves MM, Eakin EG, Winkler EA, Owen N, Healy GN (2012). Sit-stand workstations: a pilot intervention to reduce office sitting time. Am J Prev Med.

[25] van der Ploeg HP, Møller SV, Hannerz H, van der Beek AJ, Holtermann A (2015). Temporal changes in occupational sitting time in the Danish workforce and associations with all-cause mortality: results from the Danish work environment cohort study. Int J Behav Nutr Phys Act.

[26] Michaels CN, Greene AM (2013). Worksite wellness: increasing adoption of workplace health promotion programs. Health Promot Pract.

[27] Chisholm M, Russell D, Humphreys J (2011). Measuring rural allied health workforce turnover and retention: what are the patterns, determinants and costs?. Aust J Rural Health.

[28] Ware J, Kosinski M, Bjorner JB, Turner-Bowker DM, Gandek B, Maruish ME (2008). SF-36v2 health survey: administration guide for clinical trial investigators.

[29] Jenkinson C, Stewart-Brown S, Petersen S, Paice C (1999). Assessment of the SF-36 version 2 in the United Kingdom. J Epidemiol Community Health.

[30] Australian Bureau of Statistics (1995). National health survey SF-36 population norms Australia.

[31] Hagstromer M, Oja P, Sjostrom M (2006). The International Physical Activity Questionnaire (IPAQ): a study of concurrent and construct validity. Public Health Nutr.

[32] Craig CL, Marshall AL, Sjostrom M, Bauman AE, Booth ML, Ainsworth BE (2003). International physical activity questionnaire: 12-country reliability and validity. Med Sci Sports Exerc.

[33] Criniere L, Lhommet C, Caille A, Giraudeau B, Lecomte P, Couet C (2011). Reproducibility and validity of the French version of the long international physical activity questionnaire in patients with type 2 diabetes. J Phys Act Health.

[34] Kurtze N, Rangul V, Hustvedt BE (2008). Reliability and validity of the international physical activity questionnaire in the Nord-Trondelag health study (HUNT) population of men. BMC Med Res Methodol.

[35] Bond DS, Phelan S, Wolfe LG, Evans RK, Meador JG, Kellum JM (2009). Becoming physically active after bariatric surgery is associated with improved weight loss and health-related quality of life. Obesity (Silver Spring).

[36] Cloix L, Caille A, Helmer C, Bourdel-Marchasson I, Fagot-Campagna A, Fournier C (2015). Physical activity at home, at leisure, during transportation and at work in French adults with type 2 diabetes: the ENTRED physical activity study. Diabetes Metab.

[37] Nakamura PM, Teixeira IP, Smirmaul BPC, Sebastião E, Papini CB, Gobbi S (2014). Health related quality of life is differently associated with leisure-time physical activity intensities according to gender: a cross-sectional approach. Health Qual Life Outcomes.

[38] Jurakic D, Golubic A, Pedisic Z, Pori M (2014). Patterns and correlates of physical activity among middle-aged employees: a population-based, cross-sectional study. Int J Occup Med Environ Health.

[39] Prince SA, Adamo KB, Hamel ME, Hardt J, Connor Gorber S, Tremblay M (2008). A comparison of direct versus self-report measures for assessing physical activity in adults: a systematic review. Int J Behav Nutr Phys Act.

[40] Shephard RJ (2003). Limits to the measurement of habitual physical activity by questionnaires. Br J Sports Med.

[41] Ryde GC, Brown HE, Gilson ND, Brown WJ (2014). Are we chained to our desks? Describing desk-based sitting using a novel measure of occupational sitting. J Phys Act Health.

[42] Tudor-Locke C, Leonardi C, Johnson WD, Katzmarzyk PT (2011). Time spent in physical activity and sedentary behaviors on the working day: the American time use survey. J Occup Environ Med.

[43] Moreno-Franco B, Penalvo JL, Andres-Esteban EM, Malo S, Lallana MJ, Casasnovas JA (2015). Association between daily sitting time and prevalent metabolic syndrome in an adult working population: the AWHS cohort. Nutr Hosp.

[44] van Uffelen JG, Wong J, Chau JY, van der Ploeg HP, Riphagen I, Gilson ND (2010). Occupational sitting and health risks: a systematic review. Am J Prev Med.

[45] Pulsford RM, Stamatakis E, Britton AR, Brunner EJ, Hillsdon M (2015). Associations of sitting behaviours with all-cause mortality over a 16-year follow-up: the Whitehall II study. Int J Epidemiol.

[46] Carter SE, Jones M, Gladwell VF (2015). Energy expenditure and heart rate response to breaking up sedentary time with three different physical activity interventions. Nutr Metab Cardiovasc Dis.

[47] Barwais FA, Cuddihy TF, Tomson LM (2014). Adult total wellness: group differences based on sitting time and physical activity level. BMC Public Health.

[48] Healy GN, Winkler EAH, Owen N, Anuradha S, Dunstan DW (2015). Replacing sitting time with standing or stepping: associations with cardio-metabolic risk biomarkers. Eur Heart J.

[49] Chu AH, Ng SH, Tan CS, Win AM, Koh D, Muller-Riemenschneider F (2016). A systematic review and meta-analysis of workplace intervention strategies to reduce sedentary time in white-collar workers. Obes Rev.

[50] Sigmundova D, Sigmund E, Hamrik Z, Kalman M, Pavelka J, Fromel K (2015). Sedentary behaviour and physical activity of randomised sample of Czech adults aged 20–64 years: IPAQ and GPAQ studies between 2002 and 2011. Cent Eur J Public Health.

[51] Mansoubi M, Pearson N, Biddle SJ, Clemes SA (2015). Using sit-to-stand workstations in offices: Is there a compensation effect?. Med Sci Sports Exerc.

[52] O’Donovan G, Blazevich AJ, Boreham C, Cooper AR, Crank H, Ekelund U (2010). The ABC of physical activity for health: a consensus statement from the British association of sport and exercise sciences. J Sports Sci.

[53] van der Ploeg HP, Chey T, Korda RJ, Banks E, Bauman A (2012). Sitting time and all-cause mortality risk in 222 497 Australian adults. Arch Intern Med.

[54] Gebel K, Ding D, Chey T, Stamatakis E, Brown WJ, Bauman AE (2015). Effect of moderate to vigorous physical activity on all-cause mortality in middle-aged and older Australians. JAMA Intern Med.

[55] Stephens SK, Winkler EAH, Trost SG, Dunstan DW, Eakin EG, Chastin SFM (2014). Intervening to reduce workplace sitting time: how and when do changes to sitting time occur?. Br J Sports Med.

[56] Graves L, Murphy R, Shepherd SO, Cabot J, Hopkins ND (2015). Evaluation of sit-stand workstations in an office setting: a randomised controlled trial. BMC Public Health.

[57] Chau JY, Daley M, Dunn S, Srinivasan A, Do A, Bauman AE (2014). The effectiveness of sit-stand workstations for changing office workers’ sitting time: results from the Stand@Work randomized controlled trial pilot. Int J Behav Nutr Phys Act.

[58] Miyachi M, Kurita S, Tripette J, Takahara R, Yagi Y, Murakami H (2015). Installation of a stationary high desk in the workplace: effect of a 6-week intervention on physical activity. BMC Public Health.

[59] Dunstan DW, Kingwell BA, Larsen R, Healy GN, Cerin E, Hamilton MT (2012). Breaking up prolonged sitting reduces postprandial glucose and insulin responses. Diabetes Care.

[60] Puig-Ribera A, McKenna J, Gilson N, Brown WJ (2008). Change in work day step counts, wellbeing and job performance in Catalan university employees: a randomised controlled trial. Promot Educ.

[61] Thomley BS, Ray SH, Cha SS, Bauer BA (2011). Effects of a brief, comprehensive, yoga-based program on quality of life and biometric measures in an employee population: a pilot study. Explore (NY).

[62] Kurtze N, Rangul V, Hustvedt B-E (2008). Reliability and validity of the international physical activity questionnaire in the Nord-Trøndelag health study (HUNT) population of men. BMC Med Res Method.

[63] Racette SB, Deusinger SS, Inman CL, Burlis TL, Highstein GR, Buskirk TD (2009). Worksite Opportunities for Wellness (WOW): effects on cardiovascular disease risk factors after 1 year. Prev Med.

[64] Shanafelt TD, Balch CM, Bechamps GJ, Russell T, Dyrbye L, Satele D (2009). Burnout and career satisfaction among American surgeons. Ann Surg.

[65] Hadgraft NT, Lynch BM, Clark BK, Healy GN, Owen N, Dunstan DW (2015). Excessive sitting at work and at home: correlates of occupational sitting and TV viewing time in working adults. BMC Public Health.

[66] Gilson ND, Ainsworth B, Biddle S, Faulkner G, Murphy MH, Niven A (2009). A multi-site comparison of environmental characteristics to support workplace walking. Prev Med.

[67] Swartz AM, Rote AE, Welch WA, Maeda H, Hart TL, Cho YI (2014). Prompts to disrupt sitting time and increase physical activity at work, 2011–2012. Prev Chronic Dis.

[68] Sebastiao E, Gobbi S, Chodzko-Zajko W, Schwingel A, Papini CB, Nakamura PM (2012). The International Physical Activity Questionnaire-long form overestimates self-reported physical activity of Brazilian adults. Public Health.

